# Outcomes of laparoscopic ventral mesh rectopexy versus trans-vaginal repair in management of anterior rectocele, a randomized controlled trial

**DOI:** 10.1007/s10151-025-03145-z

**Published:** 2025-05-27

**Authors:** A. Sanad, A. Sakr, H. Elfeki, W. Omar, W. Thabet, E. Fouda, E. Abdallah, S. A. Elbaz

**Affiliations:** 1 Colorectal Surgery Unit, Department of Surgery, Mansoura University Hospitals, Mansoura, Egypt; 2 Department of General Surgery, King Khalid University, Abha, Saudi Arabia

**Keywords:** Rectocele, Transvaginal repair, Ventral mesh rectopexy, Functional outcome, Constipation

## Abstract

**Background:**

Anterior rectocele is one of the most common colorectal problems with symptoms of obstructed defecation or rectal emptying difficulties. The aim of this study is to compare the outcomes of laparoscopic ventral mesh rectopexy (LVMR) and transvaginal repair (TVR) for symptomatic anterior rectocele.

**Methods:**

This is a prospective randomized controlled trial conducted with 40 women. Patients were randomized into two groups. LVMR was done in the first group, whereas the second group underwent TVR. Patient outcomes were compared regarding improvement in constipation using the Cleveland Clinic Constipation (CCC) score and sexual-related quality of life score using the short form of the Pelvic Organ Prolapse/Urinary Incontinence Sexual Questionnaire (PISQ-12) at 6- and 12-month follow-up.

**Results:**

Forty females were enrolled in this trial. Each group comprised 20 patients. Preoperatively, the CCC score was 17 ± 2.8 in the LVMR group vs. 17.3 ± 2 in the TVR group (*P* = 0.278). A significant decrease in the constipation score was recorded in each group at 6 and 12 months after surgery. Regarding sexual function, the mean PISQ-12 score at 6 months was 32 ± 3.9 for LVMR vs. 35 ± 1.4 for TVR, *P* < 0.001), while at 12 months no difference was noted between the two groups. However, each group showed significant improvement in the PISQ-12 score at 6- and 12-month follow-up.

**Conclusion:**

Comparable results were noted for LVMR and TVR in management of anterior rectocele. Obstructive defecation symptoms and sexual function showed significant improvement after 1 year of follow-up. Nevertheless, long-term follow-up is needed.

**Clinical trial registration:**

The study was registered in the clinical trials registry with registration number NCT06633172.

## Introduction

Rectocele is the protrusion of the anterior wall of the rectum into the vaginal lumen through the rectovaginal fascia and posterior vaginal wall. Symptomatic rectocele affects postmenopausal women and causes obstructed defecation syndrome (ODS) [1]. Rectocele is a common problem that affects about two-thirds of multiparous women to different degrees with or without symptoms. Vaginal delivery was reported to be associated with anterior rectocele [2]. Nonetheless, 12% of nulliparous women may have rectocele because of congenital defective weakness in the rectovaginal septum [3].

Significant rectal emptying difficulties, straining at defecation, manually assisted defecation, the need for perineal or vaginal digitation, and local symptoms such as vaginal bulging and pelvic heaviness in 30–70% of cases have been described as symptoms of rectocele [4].

Constipation can be managed with dietary measures, laxatives, and biofeedback training, which can be beneficial for patients with modest symptoms [5]. Surgical treatment is recommended if conservative treatment fails to alleviate symptoms [6]. However, some patients may be left with constipation, fecal incontinence, incomplete bowel evacuation, or sexual dysfunction despite correction of the anatomical defect. Selection of patients for surgical intervention for symptomatic rectocele remains a matter of debate [7].

There is still controversy regarding the choice between abdominal approaches and the transanal, transperineal, and transvaginal approaches as the optimal surgical method to treat complex rectocele. While the latter is preferred by gynecologists, the former has increased in popularity among colorectal surgeons, aided in part by the growing interest in minimally invasive surgery [8]. To the best of our knowledge, this is the first prospective randomized study comparing both techniques, laparoscopic ventral mesh rectopexy (LVMR) and transvaginal repair (TVR); no long-term follow-up data comparing these techniques are available in the literature.

This study aimed to evaluate the outcomes of LVMR compared with TVR for anterior rectocele regarding improvement in constipation score and sexual-related quality of life, surgical outcomes, and postoperative complications.

## Methods

### Study design

This is a single-center, prospective randomized controlled trial conducted on two groups of female patients with anterior rectocele. The study was conducted at a colorectal surgical unit of the general surgery department of Mansoura University Hospital, Egypt, between April 2021 and April 2023. Ethical approval for the study was obtained from the Institutional Review Board (IRB), Faculty of Medicine, Mansoura University, no. MD.21.05.479. Written informed consent forms were obtained from eligible patients who agreed to participate in the study. The study was retrospectively registered (no. NCT06633172) in clinical trials.

### Eligibility criteria

Female patients aged between 30 and 60 years presenting with symptomatic rectocele with failed conservative treatments were included. Patients were deemed eligible if they had anterior rectocele > 3 cm in size with retention of the contrast in the rectocele on defecography with the following manifestations: excessive straining, sense of incomplete evacuation, need for digital manipulation during defecation, or dyspareunia. We excluded patients who had significant urinary manifestations due to anterior vaginal wall prolapse, recurrent rectocele, complete external rectal prolapse, other causes of ODS like isolated anismus, connective tissue disease, slow-transit constipation, fecal incontinence (FI), or abnormal thyroid function.

### Random sequence generation and blinding

**Fig. 1:**
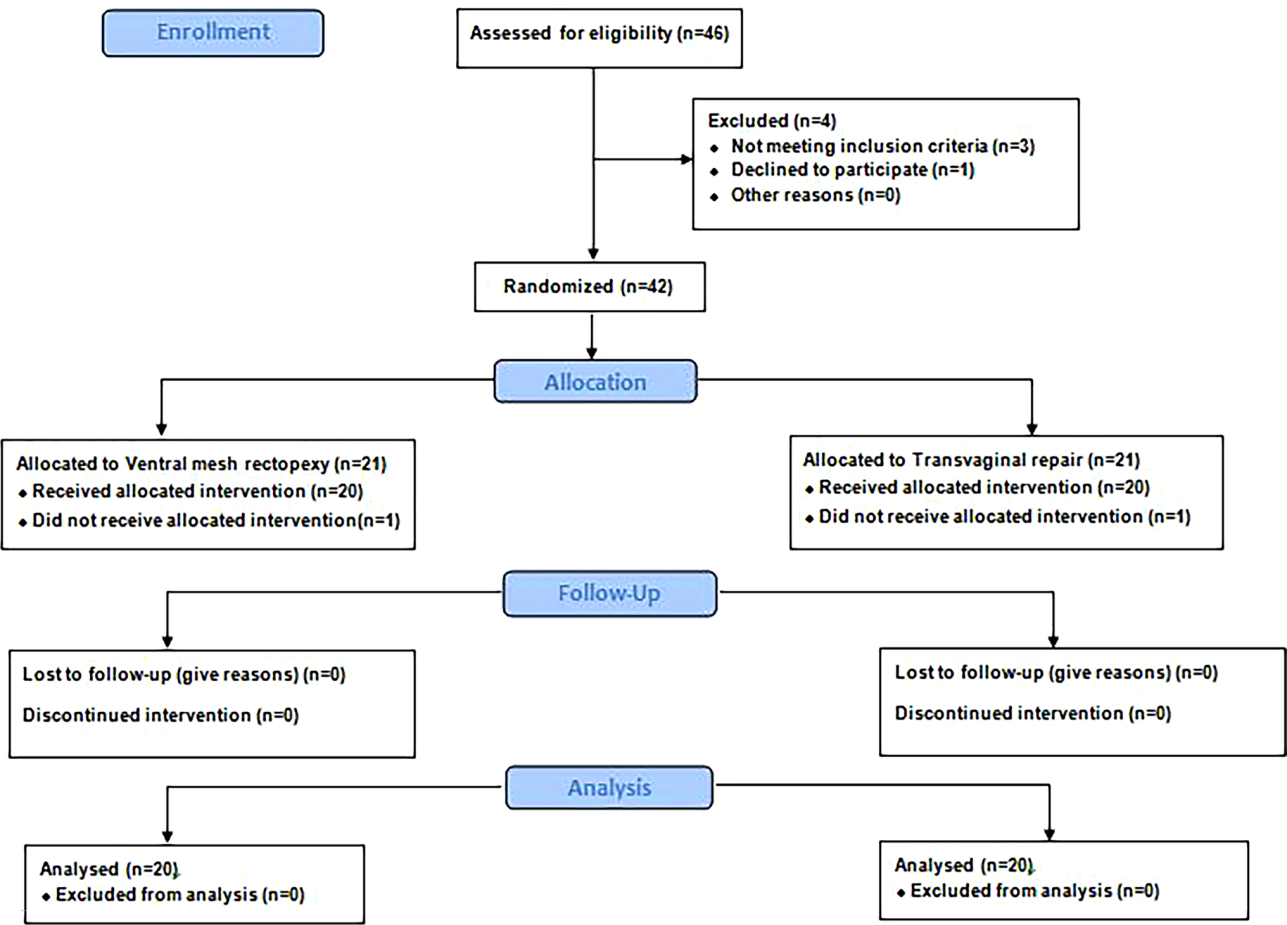
Consort flow chart

Patients were randomized to one of two equal groups: group I: LVMR, group II: TVR. Randomization was done by the sealed envelope method using randomization software (www.randomization.com) Investigators were blinded for the intervention (Fig.1)

.

## Preoperative assessment

Patients with symptomatic rectocele were assessed carefully by taking a detailed history of their complaint, associated medical conditions, bowel habits, defecation symptoms, previous treatments, and previous anal or pelvic surgeries in addition to the detailed obstetric and gynecologic history.

Functional assessment of patients was done using the Cleveland Clinic Constipation (CCC) score [9] and the short form of the Pelvic Organ Prolapse/Urinary Incontinence Sexual Questionnaire (PISQ- 12) [10]. The CCC score comprises eight questions, and the score ranges from 0 to 30 with high scores representing severe symptoms. PISQ-12 measures three domains, behavioral-emotive, physical, and partner related. Higher scores indicate better sexual function.

A thorough clinical examination was done to exclude patients with other causes of constipation and to assess the perineum carefully for any abnormalities or scars from previous surgeries. Digital rectal examination was done to detect any stricture, spasm, tenderness, mass, or retained fecal matter. Patients were investigated with colon transit time study to exclude slow-transit constipation and defecography to quantify the size of rectocele and associated abnormalities.

### Preoperative management

Patients were informed about the surgical details and its possible benefits and complications. Patients received antimicrobial prophylaxis in the form of third-generation cephalosporin and metronidazole after induction of anesthesia.

### Surgical technique

Patients underwent rectocele repair by either LVMR in group I or TVR in group II.

### LVMR technique

**Fig. 2:**
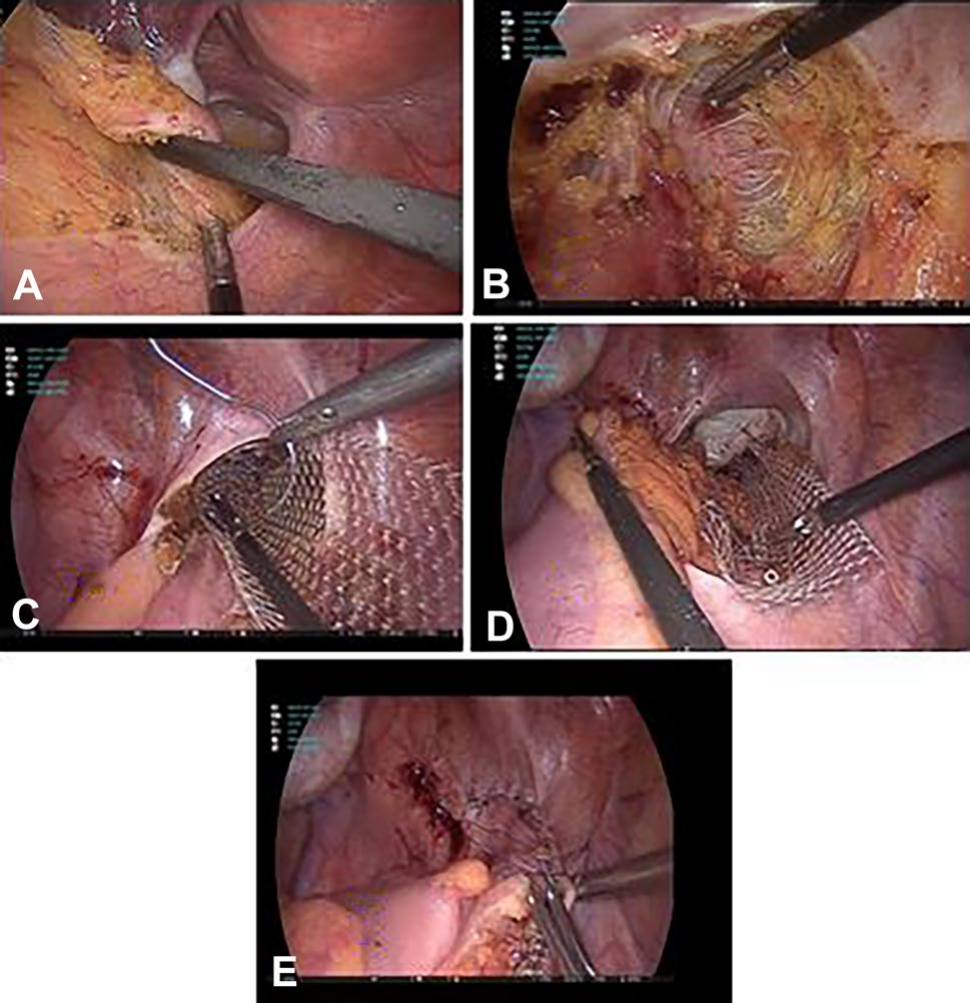
Laparoscopic ventral mesh rectopexy steps. **A** Medial peritoneal incision. **B** Complete dissection of the rectovaginal septum. **C** Mesh fixation to the rectum. **D** Mesh fixation to the sacral promontory. **E** Closure of the peritoneal incision

The LVMR procedure was performed in accordance with the original technique described by D’Hoore and colleagues [11]. Under general anesthesia and with the patients placed in a Lloyd-Davies position with steep reversed Trendelenburg position, the peritoneum was incised starting at the sacral promontory, and the dissection was extended downwards in a reversed J form over the deepest part of the pouch of Douglas . The rectovaginal septum was opened broadly down to the pelvic floor. Rectopexy was performed using a 3 × 15-cm strip of a soft, large-pore, monofilament polypropylene mesh. The mesh was affixed to the ventral aspect of the distal rectum using 2–0 polyester suture. The mesh was then fixed to the lateral seromuscular border of the rectum and pelvic floor on both sides of the rectum using 2–0 polyester sutures. The mesh was then affixed upon the sacral promontory using either 2–0 polyester sutures or 5-mm permanent tacks. Finally, the peritoneum was closed over the mesh using 2–0 spiral knotless polydioxanone monofilament suture (Fig. 2)

### TVR technique

**Fig. 3:**
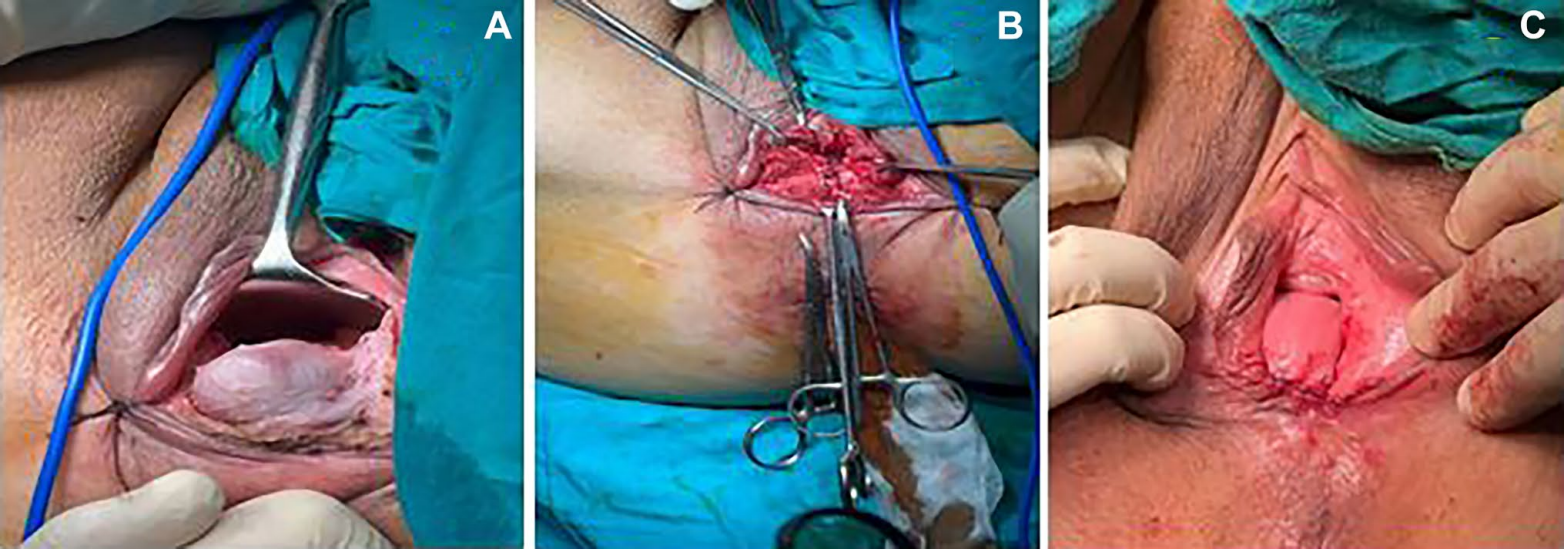
Transvaginal repair steps. **A** Complete dissection of the rectovaginal plane. **B** Midline plication of the rectovaginal septum. **C** Closure of the vaginal incision

With patients under spinal anesthesia in the lithotomy position, adrenalized saline was injected along the whole length of the rectocele. A simple transverse incision was made. Then, dissection of the vaginal epithelium from the underlying fibromuscular layer was carried out and was extended superiorly up to 1 cm above the upper aspect of the defect in the rectovaginal septum, laterally to the medial aspect of the levator ani muscles, and inferiorly down to the perineal body. The repair was performed according to the technique described by Maher et al. [12]. Using a 2–0 braided polyglactin suture, the rectovaginal fascia was plicated with interrupted multiple-bite simple stitches. Unlike the classic technique, plication of the levator ani muscles to the midline was not performed to avoid dyspareunia. Subsequently, the continuity of the vaginal epithelium was restored using the 2–0 braided polyglactin suture after careful hemostasis (Fig. 3)

### Postoperative care

Patients were monitored to detect early complications after surgery. Oral feeding was resumed on the same day as the operation, and analgesia with non-steroidal anti-inflammatory medications was administered on patient demand. Patients were discharged home on laxatives for 1 month after the procedure. Patients were advised to increase fluid intake and dietary fiber, avoid excessive straining, and adhere to the follow-up schedule. Patients in the transvaginal group were prescribed metronidazole vaginal suppositories and were advised to maintain local perineal hygiene. Patients in both groups were advised to avoid sexual intercourse for 6 weeks after the procedure.

### Follow-up

Patients were followed up in the outpatient clinic weekly for the 1st month, then every 2 weeks in the 2nd month, and at 3, 6, and 12 months postoperatively. During the follow-up visits, patients were examined to detect any complications and assess the state of the wound in terms of wound healing, wound disruption, collection, hematoma, and surgical site infection (SSI). Symptomatic improvements in constipation and sexual symptoms were evaluated using CCC scores and PISQ-12 at 6 and 12 months postoperatively.

### Study outcomes

The primary outcome of the study was the absolute decline in CCC score. Secondary outcomes included improvement in sexual function (PISQ- 12), operative time, time to healing, and postoperative complications.

### Sample size calculation

The sample size was calculated using sample size calculation software (https://clincalc.com/stats/samplesize.aspx) based on the primary outcome of the study (absolute decline in CCC scores). Based on a previous retrospective study, Abdelnaby et al. [13] reported a decline of about eight CCC score points in the LVMR group and of about five in the TVR group, with a standard deviation (SD) of 3. To achieve a study power of 85% with alpha set at 5%, a minimum of 36 patients, equally divided into two groups, was needed. To compensate for loss of follow-up, an ultimate sample size of 40 patients needed to be included in the study.

### Statistical analysis

Statistical analysis of data was performed using SPSS software, version 23 (Bristol, UK). For quantitative variables, continuous variables were described as mean ± SD and discrete variables as median and range. Categorical variables were reported using numbers and proportions. Student's *t*-test for paired samples was used to detect differences in the means of continuous variables. Fisher's exact test or chi-square test was used to process categorical variables. Mann-Whitney U-test was used to detect differences in the medians of discrete variables. *P* values < 0.05 were considered significant.

## Results

Forty female patients with a median age of 45 (range 35–61) years were included in this study. All patients presented with obstructed defecation with a median duration of 4 (range 2– 10) years. Regarding obstetric history, 2 (5%) had single vaginal deliveries, 10 (25%) had 2 vaginal deliveries, and 28 (70%) had > 2 vaginal deliveries. There were no significant differences between the two groups regarding patients’ characteristics, duration of symptoms, and size of rectocele. Patients in the LVMR group had a significantly longer hospital stay and operative time than patients in the TVR group (*P* < 0.001).

The only postoperative complications recorded were one patient in the LVMR group with postoperative ileus, which resolved early on the 3rd postoperative day with conservative treatment, and one patient with postoperative chest infection who responded well to intravenous antibiotics. No mesh-related complications were reported in the LVMR group at 1-year follow-up. Neither new-onset constipation nor new-onset pelvic pain was recoded after LVMR.

In the TVR group, two patients developed postoperative urinary tract infections (UTI), while one woman developed surgical site infection with incomplete disruption of the suture line, which responded well to conservative measures without requiring surgical intervention. At 1-year follow-up, no patients reported recurrence of symptoms in either group. There were no significant differences between the two groups regarding postoperative complications (Table 1).

**Table 1:** Patients' demographic data

Variable	LVMR (*n* = 20)	TVR (*n* = 20)	*P* value
Age(years) (median ± range)	44(35–60)	43(38–61)	0.753
BMI, kg\m² (mean ± SD),	30.5 ± 3.4	30.7 ± 3.9	0.864
Duration of symptoms (years) (median ± range)	4 (2–10)	4 (2–8)	1
Number of vaginal deliveries (median ± range)	2.5 (1–5)	3 (1–5)	0.309
Size of rectocele in defecography in cm (mean ± SD)	5 ± 0.9	5.4 ± 1	0.253
Hospital stay (days)(mean ± SD)	4 ± 0.6	1.55 ± 0.6	**< 0.001**
Operative time, minutes (mean ± SD)	208 ± 22.1	90 ± 15.2	**< 0.001**
Minor complications (*n*%)	2(10%)	3(15%)	0.547
Major complication (*n*%)	0	0	1.0

Bold values indicate statistical significance (*P* <values)

*LVMR* laparoscopic ventral mesh rectopexy, *TVR* transvaginal repair, *BMI* body mass index

### Postoperative change in constipation score

The CCC score was 17 ± 2.8 in the LVMR group vs. 17.3 ± 2 in the TVR group with no significant difference between the two groups preoperatively. A significant decrease in the constipation score was recorded in each group at 6 and 12 months after surgery. Improvement in CCC score was higher in the TVR group at 6-month follow-up, while at 1 year it was better in the LVMR group. However, the difference was not statistically significant (Table 2).

**Table 2:** Postoperative change in constipation score in the two groups

Variable	LVMR group (*n* = 20)	TVR group (*n* = 20)	*P*_1_ value
Mean preoperative CCC score	17 ± 2.8	17.3 ± 2	0.278
Mean CCC score at 6 months	11.6 ± 1.4	11 ± 1.7	0.235
Mean CCC score at 12 months	10.5 ± 2.1	11.2 ± 1.2	0.112
P_2_ value	**< 0.001**	**0.005**	

Bold values indicate statistical significance (*P* <values)

*LVMR* laparoscopic ventral mesh rectopexy, *TVR* transvaginal repair, *CCC* Cleveland Clinic Constipation score

**P2 value: difference between mean preoperative and postoperative score at 6 and 12 months in each group, respectively

### Postoperative change in sexual function

The study included 23 (57%) sexually active women (12 in LVMR vs. 11 in TVR). Six (26%) refused to complete the PISQ-12 questionnaire because of embarrassment. Finally, the PISQ-12 score was calculated for only 17 females (10 in LVMR vs. 7 in TVR). Both groups had comparable preoperative PISQ-12 scores. Improvement was reported in both groups at 6-month follow-up, but it was statistically significant only in the TVR group. Moreover, the TVR group showed better improvement in the PISQ-12 score at the same follow-up period (*P* =  < 0.001). At 12-month follow-up, both groups achieved significant improvement in PISQ-12 score (*P* = 0.005). However, no difference was noted between the two groups at the same follow-up period (*P* = 0.244) (Table 3).

**Table 3:** Postoperative change in sexual-related quality of life in the two groups

Variables	LVMR group (n=10)	TVR group (n=7)	*P*_1_ value
Mean preoperative PISQ-12 score	25.6 ± 3.3	25 ± 2.2	0.678
Mean PISQ-12 score at 6 months	32 ± 3.9	35 ± 1.4	**< 0.001**
Mean PISQ-12 score at 12 months	35 ± 3.6	35 ± 1.4	0.244
*P*_2_ value	**0.005**	**0.005**	

Bold values indicate statistical significance (*P* <values)

*LVMR* laparoscopic ventral mesh rectopexy, *TVR* transvaginal repair, *PISQ_12 score* Pelvic Organ Prolapse/Urinary Incontinence Sexual Questionnaire

**P2 value: difference between mean preoperative and postoperative score at 6 and 12 months in each group, respectively

### Dyspareunia assessment as a separate item

In the LVMR group, six (50%) patients complained of dyspareunia. Three (50%) reported significant improvement, while the other three showed neither improvement nor worsening. New-onset dyspareunia was recorded in two patients at 3-month follow-up; however, one reported improvement at 6- and 12-month follow-up. In the TVR group, seven (63%) patients complained of dyspareunia. Four (57%) reported improvement, while the other three showed neither improvement nor worsening. None of patients in the TVR group reported new-onset dyspareunia. We attributed the absence of new-onset dyspareunia in the TVR group to non-plication of the levator muscle during the repair. We tried to address whether there was any association among the rectocele size, number of vaginal deliveries, and duration of ODS symptoms due to rectocele and preoperative, postoperative, and new-onset dyspareunia, but we could not determine any significance because of the small sample size of patients who were not embarrassed.

## Discussion

The current study compared the efficacy of LVMR and TVR in improving constipation and sexual-related symptoms of anterior rectocele. Despite the lack of randomized controlled trials comparing the different surgical techniques for rectocele management, the present trial is the third conducted in our unit comparing the two different techniques.

In the first study, Farid et al. compared three groups (transperineal, transperineal with levatorplasty, and transanal) with 16 patients in each group and concluded that transperineal repair of rectocele is superior to transanal repair in terms of both structural and functional outcomes. Levatorplasty improves functional outcomes, but patients may suffer from dyspareunia [4]. In the second trial, Balata et al. compared two groups of 32 patients (transvaginal and transperineal) and reported the superiority of transvaginal repair regarding improvement in constipation score and the sexual-related quality of life [14].

TVR was gynecologists' procedure of choice. This technique has historically provided good functional and anatomical results, ranging from 76–96%. However, many studies have reported a high rate of dyspareunia, between 20–50%, which may be attributed to too tight levator plication, causing vaginal narrowing [15, 16]. This led to the evolution of a modified rectocele repair, where instead of plication of the levator muscles in the midline, discrete facial defects in the rectovaginal septum were closed. Several studies noted improvement in sexual dysfunction ranging from 66–92% of patients [17–19].

In the current trial, four of seven patients (57%) reported improvement of the preoperative dyspareunia, while the other three showed neither improvement nor worsening. Moreover, no new-onset dyspareunia was reported. This was consistent with the literature, emphasizing that levatorplasty increases the risk of de novo dyspareunia. Moreover, significant improvement in PISQ-12 scores was recorded at 6 and 12 months in the TVR group. A recent systematic review analyzing 46 studies reported six surgery types for posterior compartment prolapse and concluded that transvaginal repair should be the first choice procedure, although the results may decrease over time [20].

Concerning the long-term functional results of the transvaginal approach, few data are available in the literature. A study by Chung et al. reported deterioration of long-term functional outcomes after the transvaginal or transanal approach despite satisfactory results at 12 months, with > 75% of patients improved; there was a decrease in satisfaction at 50 (36–59) months, regardless of the approach [21]. In the present study, significant improvement in CCC scores was noted after transvaginal repair. At 1-year follow-up, CCC scores decreased by about 6 points from 17.3 ± 2 preoperatively to 11.2 ± 1.2 postoperatively.

LVMR was first introduced by D’Hoore in 2004; it was initially developed for patients with external rectal prolapse [11]. A consensus from a panel of specialists concluded that high-grade internal rectal prolapse (IRP) and/or complex rectocele with persistent ODS could be a relative indication for LVMR, even though it is still debated [22]. Theoretically, compared to other methods, the laparoscopic method for complex rectocele provides a number of benefits. First, it avoids the transperineal and transvaginal route, which reduces the dyspareunia frequently related to these approaches. Second, avoiding transanal dilatation lowers the likelihood of incontinence. Third, it enables the surgeon to manage multiple pathologies concurrently given that many patients exhibit multiple organ prolapses.

Notably, the correction of anatomy, confirmed by postoperative defecography, does not necessarily result in meaningful symptomatic relief [23]. This may explain the variety of outcomes regarding ODS alleviation described in the literature; a recent systematic review reported overall postoperative improvement of obstructive defecation in 55–86% of patients, and the observed CCC score improvement was between 3.1 and 9 points across studies on LVMR for IRP and/or rectocele [24]. In the present study, significant improvement in CCC score was reported after LVMR. At 1-year follow-up, the CCC score decreased by about 6.5 points from 17 ± 2.8 preoperatively to 10.5 ± 2.1 postoperatively.

Complications following LVMR need to be given extra attention. Badrek-Al Amoudi et al. reported a list of serious side effects (rectal stricture, erosion, pelvic pain) occurring in patients after ventral mesh rectopexy handled in a tertiary referral center [25].

Mesh-related complications remain a concern, although these did not occur in our study. Eighteen (4.8%) participants in a retrospective study with a large cohort of 919 patients and a median follow-up of 33 months experienced mesh-related problems [26]. It has been suggested that biologic mesh lowers the risk of mesh erosion. Mesh erosion was reported in a multicenter study of 2203 patients after LVMR, occurring in 2.4% after using synthetic mesh and 0.7% after using biologic mesh, with a median of 23 months [27]. A recently published review reporting the incidence of mesh-related complications after LVMR reported that mesh-related erosion after LVMR is more frequent after synthetic mesh placement, even though the reported incidence rate for both synthetic and biologic meshes is low (synthetic, 1.87%; biologic, 0.22% respectively) [28].

Regarding sexual function after LVMR, in a recently published critical appraisal of the increasing practice of LVMR based on low-level evidence, the authors argued that high-level evidence needs to be generated and attention in further studies should focus not only on restoration of bowel function but also on sexual problems [29]. In the literature, there is a paucity of reports on sexual function with different outcomes. Worsening of sexual problems was reported by Horisberger et al. [30], with more than half of the women showing an impairment of their sex life after the operation and fewer than 50% describing an improvement. They concluded that, after ventral mesh rectopexy for obstructive defecation, there was encouraging improvement in constipation and quality of life. However, the effects on sexual life vary; while some patients experience improvements, a significant number report a negative impact. On the other hand, two French studies reported the positive effect of LVMR on the sexual problems in patients with complex rectocele as there was a significant improvement of dyspareunia in 85% of patients who responded to the brief Index of Sexual Functioning for Women (BISF-W) questionnaire. In addition, no de novo dyspareunia was observed [8, 31].

Another major concern is the quality of life after this kind of surgery. A Chinese study emphasized the improvement of patients’ quality of life in all four subsets of the Patient Assessment of Constipation Quality of Life Questionnaire (PAC-QOL): Three showed statistical significance (physical discomfort, worries and concerns, satisfaction) after LVMR for obstructive defecation in patients with overt pelvic structural abnormalities [32]. In a recent retrospective study, Abdelnaby et al. reported a significant improvement in CCC score, PISQ-12, and PAC-QOL in 72 patients with complex rectocele who underwent LVMR compared with 159 patients who underwent transvaginal posterior colporrhaphy [13].

In our collective data, the improvement in PISQ-12 in the LVMR group did not reach a significant level during the first 6 months of follow-up but showed a significant improvement at 12-month follow-up. In addition, three of six patients who had preoperative dyspareunia improved postoperatively, while the other three did not show any improvement or worsening. Moreover, new-onset dyspareunia was recorded in two patients at 3-month follow-up; however, one reported improvement at 6- and 12-month follow-up.

Finally, female sexual dysfunction can be caused by a variety of factors, including physical (neurologic, vascular, and muscular) and psychologic changes. Surgical trauma is just one of many potential causes. Therefore, sexual disorders are a frequently neglected issue that may have a negative impact on the patient's quality of life. Adding to the complexity of management, up to 45% of patients with sexual difficulties do not disclose it to doctors because of embarrassment, and up to 56% do not even consider it to be a surgical concern [33]. Reported causes of postoperative dyspareunia after surgery for rectocele and pelvic organ prolapse include surgical scar at the vagina or perineal area, vaginal stenosis, levator plication, mesh erosions or mesh shrinkage, and/or extensive fibrosis from the surgery or the mesh [34].

Limitations of the present study are the small sample size and the short-term follow-up. Further prospective studies with large sample size are needed to establish solid evidence.

## Conclusions

Laparoscopic ventral mesh rectopexy and transvaginal repair are effective methods for rectocele management with comparable outcomes. Obstructive defecation symptoms and sexual function showed significant improvement after 1 year of follow-up after both techniques. Patients with outlet obstructive symptoms including digitation may benefit from TVR, whereas LVMR can be offered to patients with rectorectal (internal) intussusception or mucosal prolapse together with anterior rectocele. Nevertheless, further prospective studies with long-term follow-up data are needed to establish solid evidence on the efficacy of both techniques.

## Data Availability

No datasets were generated or analysed during the current study.
